# Characterization of Necroptosis-Related Molecular Subtypes and Therapeutic Response in Lung Adenocarcinoma

**DOI:** 10.3389/fgene.2022.920350

**Published:** 2022-06-08

**Authors:** Jingchen Zhang, Xujian He, Jia Hu, Tong Li

**Affiliations:** The First Affiliated Hospital, Zhejiang University, Hangzhou, China

**Keywords:** lung adenocarcinoma, necroptosis, molecular subtypes, tumor microenvironment, therapeutic response, prognosis

## Abstract

Lung adenocarcinoma (LUAD) is one of the most common malignant tumors with high morbidity and mortality and is usually associated with therapeutic resistance and poor prognosis because of individual biological heterogeneity. There is an unmet need to screen for reliable parameters, especially immunotherapy-related biomarkers to predict the patient’s outcomes. Necroptosis is a special caspase-independent form of necrotic cell death associated with the pathogenesis, progression, and prognosis of multiple tumors but the potential connection between necroptosis-related genes (NRGs) and LUAD still remains unclear. In this study, we expounded mutational and transcriptional alterations of 67 NRGs in 522 LUAD samples and proposed a consensus-clustering subtype of these patients into two cohorts with distinct immunological and clinical prognosis characteristics. Cluster B patients were associated with a better prognosis and characterized by relatively lower expression of NRGs, higher immune scores in the tumor microenvironment (TME), more mild clinical stages, and downregulated expression of immunotherapy checkpoints. Subsequently, the NRG score was further established to predict the overall survival (OS) of LUAD patients using univariate Cox, LASSO, and multivariate Cox regression analyses. The immunological characteristics and potential predictive capability of NRG scores were further validated by 583 LUAD patients in external datasets. In addition to better survival and immune-activated conditions, low-NRG-score cohorts exhibited a significant positive correlation with the mRNA stem index (mRNAsi) and tumor mutation burden (TMB) levels. Combined with classical clinical characteristics and NRG scores, we successfully defined a novel necroptosis-related nomogram to accurately predict the 1/3/5-year survival rate of individual LUAD patients, and the potential predictive capability was further estimated and validated in multiple test datasets with high AUC values. Integrated transcriptomic analysis helps us seek vital NRGs and supplements a novel clinical application of NRG scores in predicting the overall survival and therapeutic benefits for LUAD patients.

## Introduction

Worldwide, lung cancer is one of the most common malignant tumors with high morbidity and mortality and has a poor prognosis with critical social burdens (Herbst et al., 2018). Non-small cell lung cancer (NSCLC) makes up approximately 85% of all lung cancers, and lung adenocarcinoma (LUAD) is the most common pathological subtype of NSCLC (Chen F. et al., 2022). Notably, more than 50% of LUAD patients were at advanced stages when they were clinically diagnosed and the prognosis was relatively poor with only 11–15% 5-year overall survival (OS) rate (Bade and Dela Cruz, 2020). Despite the treatment of LUAD being improved remarkably, including surgery, chemotherapy, and radiotherapy based on the clinical stages of LUAD, there is still a lack of effective curative effects for advanced LUAD treatment (Denisenko et al., 2018). At present, the clinical progress of PD-1/PD-L1 immunotherapy has brought a promising therapeutic potential for LUAD patients, especially for those resistant to conventional surgery, radiation, or chemotherapy (Saito et al., 2018). Nevertheless, in our clinical practice, even if LUAD patients were at the same pathological stages, their therapeutical response to immunotherapy might still be completely different (Skoulidis and Heymach, 2019). Therefore, there is an unmet need for screening reliable biomarkers, especially the PD-1/PD-L1 immunotherapy-related index, which could predict outcomes of LUAD patients.

Necroptosis is a special caspase-independent form of necrotic cell death characterized by cell membrane rupture and inflammatory response activation regulated by receptor-interacting protein kinase1/3 (RIPK1/3) and mixed lineage kinase domain-like pseudokinase (MLKL) (Pasparakis and Vandenabeele, 2015). Increasing pieces of evidence have indicated the double-edged sword role of necroptosis in multiple tumors. For example, necrotic tumor cells would release their contents and further activate the inflammatory and immunological response of surrounding immune cells (Krysko et al., 2017; Wang et al., 2020). On the other hand, necroptosis might also promote tumor progression and metastasis by killing normal paraneoplastic cells and leading to severe inflammatory disorders (Ando et al., 2020). Moreover, recent studies have indicated that necroptosis could create an inflammatory environment to enhance the tumor susceptibility to immune checkpoint inhibitors in drug-resistant tumors (Workenhe et al., 2020). These studies indicated the complex connection between necroptosis and LUAD, but the concrete mechanism of necroptosis in LUAD still remains unclear.

The subtype stratification of LUAD patients based on transcriptome sequencing profiles has been recognized as a novel methodology that can quickly obtain biological characteristics of subtypes and help us further identify the optimal treatment strategies for patients (Jang et al., 2020). In addition, multiple biological signatures have also been applied to explore novel molecular subtypes for the prognosis of LUAD, such as immune cell infiltration (ICI) (Li et al., 2021), autophagy (Zhang M.-Y. et al., 2021), pyroptosis (Dong et al., 2021), m6A RNA methylation (Zhou et al., 2021), and so on. However, there is still no study focusing on the role of necroptosis in the subtypes of LUAD patients. In this study, we comprehensively investigated the genetic and biological characteristics of NRGs in LUAD patients and first divided the cohorts into different subtypes based on the expression of NRGs. The clinical prognostic signatures and immunological landscape of necroptosis-related subtypes were further interpreted through survival analysis, tumor microenvironment (TME) assessments, immune cell infiltration (ICI) analysis, and immune checkpoint comparison. Subsequently, a novel parameter called NRG-score was further defined based on vital NRGs, and a valuable nomogram, combined NRG scores with some classical clinical stages, was successfully established and validated to ameliorate the prognostic stratification and promote making an appropriate therapeutic decision for LUAD patients.

## Material and Methods

### Preparation of Lung Adenocarcinoma Datasets

The public RNA-seq transcriptome datasets of 522 LUAD patients were downloaded from The Cancer Genome Atlas (TCGA) database (https://portal.gdc.cancer.gov/) with their corresponding clinical data. In addition, three external datasets of 583 LUAD patients with their prognostic information were obtained from the Gene Expression Omnibus (GEO) datasets (https://www.ncbi.nlm.nih.gov/geo/), including 176 samples in GSE42127, 226 samples in GSE31210, and 181 samples in GSE58001. The detailed information of the aforementioned datasets is shown in Supplementary Table S1. Then, all the datasets were normalized as the FPKM form for subsequent analysis and the “ComBat” algorithm of the “sva” package was applied to remove the technical biases between different datasets (Leek et al., 2012).

### Mutational Characteristics of Necroptosis-Related Signatures in Lung Adenocarcinoma

Based on the necroptosis-related dataset M24779. gmt and previous studies, a total of 67 NRGs were chosen in this study such as mixed lineage kinase domain-like pseudokinase (MLKL), receptor-interacting protein kinase 1 (RIPK1), RIPK3, and so on (Zhao Z. et al., 2021; Chen F. et al., 2022). Subsequently, we also obtained their corresponding mutation annotation format (MAF) from the UCSC Xena online platform including copy number variants (CNVs) and somatic mutation data. The “maftools” package (Mayakonda et al., 2018) was used to display the somatic mutation of NRGs, and the “RCircos” package (Zhang et al., 2013) was applied to exhibit their CNVs and locations on the respective chromosomes.

### Identification of Consensus Clusters for Lung Adenocarcinoma

Based on the expression of these NRGs, we applied the “ConsensuClusterPlus” R package (Wilkerson and Hayes, 2010) to perform the unsupervised hierarchical clustering analysis using the Euclidean distance and Ward’s linkage algorithm 1,000 repeated times. Moreover, the LUAD patients were divided into different subtypes from two to nine, and the optimal clustering subtype was further decided with the optimal consensus cumulative distribution function (CDF) curve. In addition, we conducted various comparisons among different clusters including the clinical-pathological stages, prognostic characteristics, and tumor microenvironment (TME) analysis to explore their disease characteristics. The Kaplan–Meier survival analysis was performed using the “survival” package (Therneau and Lumley, 2015) and survival curves between necroptosis subtypes were drawn by the “survminer” package (Kassambara et al., 2017).

### Immunological Characteristics of Different Clusters in Lung Adenocarcinoma

To explore the immunological characteristics of necroptosis-related clustering, we further performed a comprehensive analysis according to different immunological aspects, including immune cell infiltration (ICI) analysis, tumor microenvironment (TME) analysis, and immune checkpoint analysis. For the TME analysis, we used the ESTIMATE algorithm to calculate the stromal scores, immune scores, and tumor purity of each LUAD patient (Yoshihara et al., 2013). To quantitatively estimate the infiltration levels of immune cells in lung tissues, we applied the deconvolution algorithm of the “CIBERSORT” package with 22 different immune cells and 1,000 random permutations (Chen et al., 2018). In addition, the expression of routine immune checkpoints was compared between necroptosis-related subtypes to evaluate the potential therapeutic responses, including CTLA4, PD1/CD274, HAVCR2, PD-L1/PDCD1, and LAG3. Based on the “c2. cp.kegg.v7.5.1. symbols.gmt” datasets obtained from the MSigDB database, we further performed the gene set variation analysis (GSVA) using the “GSVA” package, and the results of immunogenic pathways were displayed in the heatmap (Hanzelmann et al., 2013).

### Establishment of the Necroptosis-Related Gene Score

To further establish a novel index reflecting the prognostic features of necroptosis-related subtypes, we performed the univariate Cox regression analysis for the overall survival (OS) of LUAD patients through the “coxph” function in the “survival” package. After filtration with the *p*-value < 0.05, the remaining NRGs were further put into the LASSO regression and multivariate Cox regression (stepwise model) in turn to obtain the corresponding regressive coefficients. The NRG score was identified based on the following formula: NRG score=Exp(Gene1)*β1+Exp(Gene2)*β2+…+Exp(Gene n)*βn, where Exp(Gene) denotes the FPKM value of each gene and β is their corresponding regression coefficient. The NRG scores of each patient were calculated separately and the subjects were divided into high and low-NRG-score subtypes according to the optimal cut-off value by the “surv_cutpoint” function of the “survminer” package (Kassambara et al., 2017). In addition, we also performed a similar comparison between high and low NRG groups including the Kaplan–Meier survival analysis, clinical stages, ICI, TME, and immune checkpoint analysis. Moreover, two external datasets, GSE126044 and GSE135222, were applied to further evaluate the therapeutic response to immunotherapy, including 16 and 27 NSCLC patients receiving anti-PD-1 therapy. Some anti-tumor drugs have been widely recommended for the chemotherapy of LUAD including Etoposide, Cisplatin, Gemcitabine, and Docetaxel, and the half-maximal inhibitory concentration (IC50) values of these drugs were calculated based on the Genomics of Drug Sensitivity in Cancer (GDSC) datasets (Yang et al., 2013). Then, we compared the levels of IC50 values between high- and low-NRG score patients, and the box diagram was drawn via the “ggpubr” package (Whitehead et al., 2019).

### Relationship Between the mRNA Stem Index, Tumor Mutation Burden, and NRG Scores

To investigate the potential prognostic characteristics of LUAD, we obtained the mRNAsi score from Tathiane’s study (Malta et al., 2018) and gained the TMB score based on the mutation data from TCGA datasets. Subsequently, we performed Spearman’s correlation analysis of NRG scores with mRNAsi and TMB scores. In addition, the stratified survival analysis was further applied to evaluate the independent prognostic capacity of NRG and TMB scores in LUAD and the mutational analysis was conducted in high- and low-NRG score subgroups, respectively.

### Identification and Validation of a Novel NRG-Related Nomogram

To validate the prognostic value of NRG scores, other three external GEO datasets were included to perform survival and ROC analyses with 1/3/5-year survival rates for LUAD patients. Then, we applied the multivariate Cox regression models (stepwise model) to construct a novel prognostic nomogram system for LUAD patients combined with NRG scores and other important clinical phenotypes, including age, clinical stages, and TNM stages. Selected variables were screened with a *p* value < 0.05 or saved based on clinical experiences, and the nomogram system was further constructed to determine the probability of 1/3/5-year survival in LUAD patients via the “rms” package. To assess and validate the prediction value of the nomogram scoring system, we further made the calibration curves with the corresponding 1-, 3-, and 5-year survival through a bootstrapping method. Moreover, time-dependent ROC and calibration curves from the other three external GEO datasets were used to estimate the nomogram for 1-, 3-, and 5-year survivals.

## Results

### Genetic Mutation and Prognostic Characteristics of NRGs in Lung Adenocarcinoma

**FIGURE 1:**
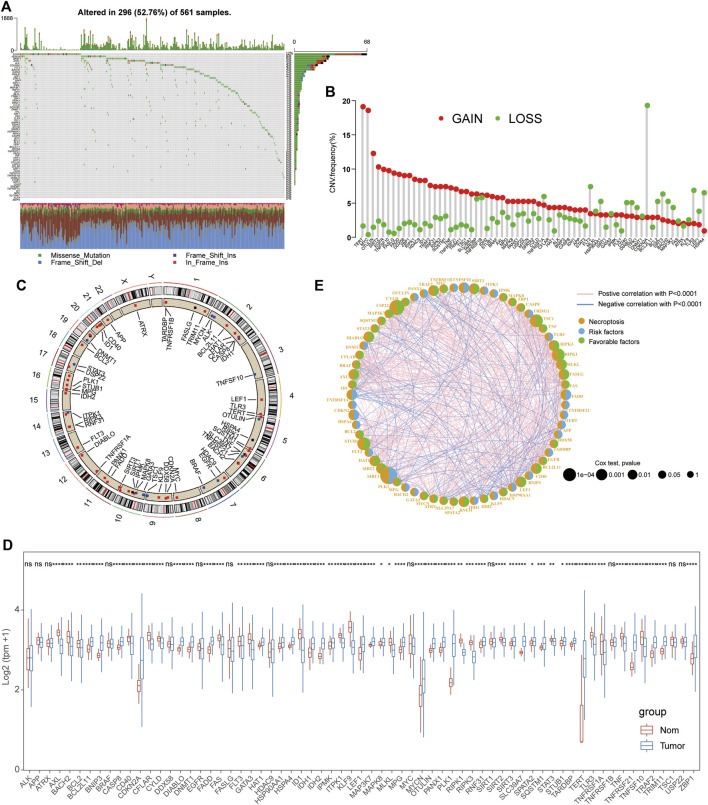
Mutational and expressional characteristics of NRGs in LUAD patients. **(A)** Waterfall figure showing the somatic mutation of 67 NRGs in LUAD; **(B–C)** Situation of CNV gain and loss of the PRGs on 23 chromosomes. **(D)** Expression of these NRGs between LUAD and the control. **(E)**. Prognostic characteristics and expressional relationship among PRGs in LUAD.

The whole workflow of this study is given in Supplementary Figure S1. A total of 67 reported NRGs were chosen to perform the genetic mutation analysis, including somatic mutation and copy number variant analyses (Supplementary Table S1). Of all 561 LUAD patients, about 52.76% samples were detected with somatic mutations and the top three genes with the most mutations were EGFR, HDAC9, and BRAF (Figure 1A). The CNV analysis revealed that most NRGs occurred due to copy number amplification (TERT and MYT) and some copy number deficiency was also identified in partial NRGs (CDKN2A) (Figure 1B; Supplementary Table S2). In addition, we also observed that the CNV of NRGs was widely distributed into multiple chromosomes with amplification or deficiency (Figure 1C), and the mutual correlation and prognostic values of NRGs in LUAD patients were displayed in a comprehensive network (Figure 1E, Supplementary Tables S4, 5). Interestingly, we also compared their expression levels between tumor and normal tissues and found that the expressional changes were complex, especially significantly upregulated genes (CDKN2A, MYCN, PLK1, and TERT) and some downregulated signatures (AXL, ID1, and TLR3) (Figure 1D, Supplementary Table S3).

### Identification of Necroptosis-Related Subtypes and Characteristics in Lung Adenocarcinoma

**FIGURE 2:**
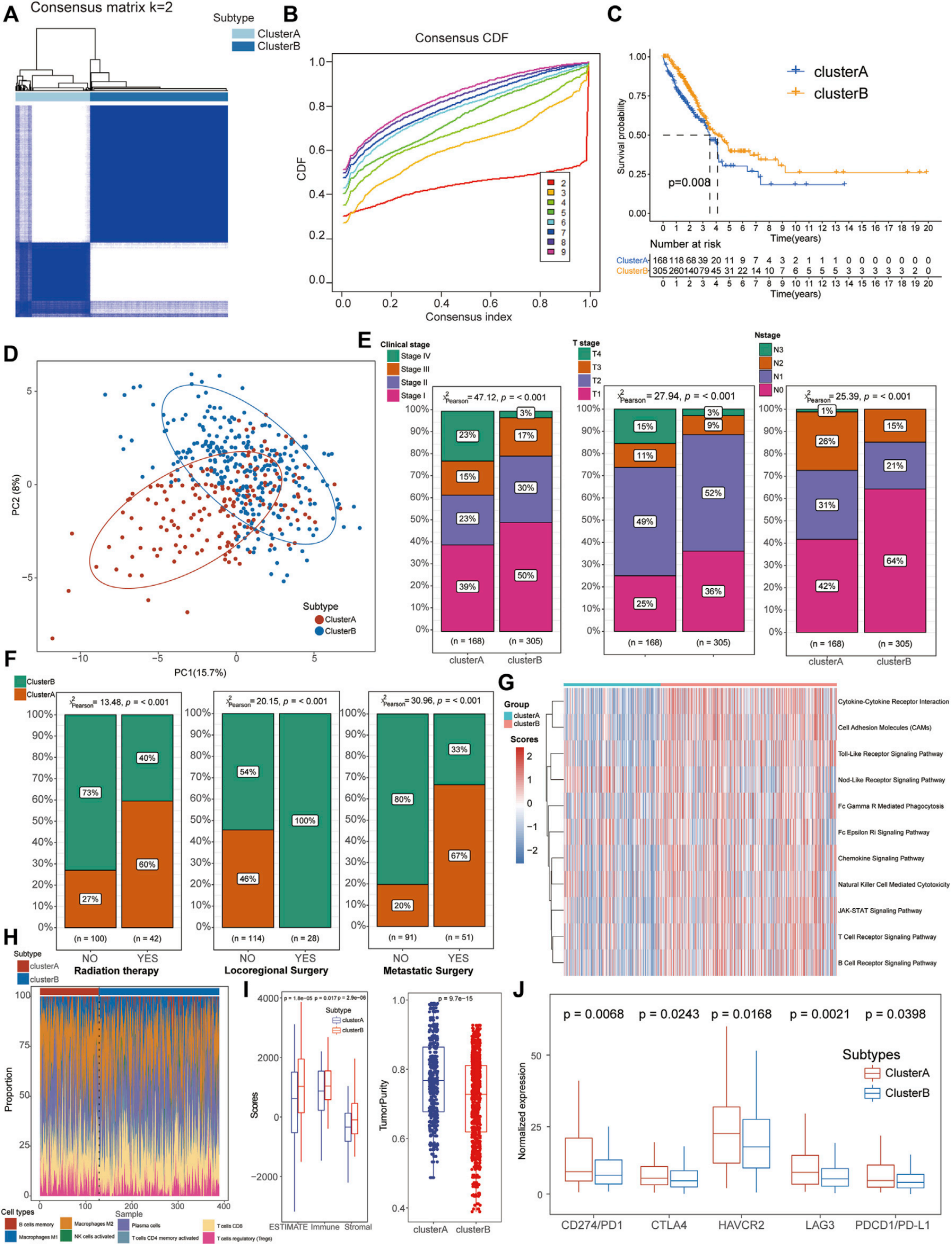
Identification of necroptosis-related molecular subtypes and immunological characteristics in LUAD. **(A,B)** Patients could be well divided into two subtypes based on CDF curves. **(C)** K–M survival analysis exhibited a better prognosis for cluster B patients than that of cluster A groups**. (D)** PCA analysis showed significant differences in the necroptosis-related transcription profiles between the two subgroups**. (E)** Cluster B patients manifested more proportion of mild clinical stages than cluster A patients. **(F)** Compared with cluster A patients, most cluster B patients accepted locoregional surgical treatment rather than metastatic surgery or radiation therapy. **(G)** GSVA demonstrated that the immune-related pathways were significantly activated in cluster B patients compared with cluster A groups. **(H)** Comparison of immune cell infiltration between the two clusters. **(I)** Higher stromal and immune scores with lower tumor purity were detected in cluster B patients based on the TME analysis. **(J)** Cluster A patients exhibited higher expression of immune checkpoints, including CD274/PD1, PDCD1/PD-L1, CTLA4, HAVCR2, and LAG3.

Based on the expression of the aforementioned 67 NRGs, an unsupervised clustering method was applied to identify the necroptosis-related subtypes of LUAD patients, and *k* = 2 was further identified as the optimal clustering model from *k* = 2 to 9 clustering according to the consensus CDF curve, for 168 patients in cluster A and 300 patients in cluster B subtypes (Figures 2A, B). The survival analysis indicated that patients from cluster B had a longer median survival time than those in cluster A subgroups (Figure 2C) and the PCA revealed that the expression of these NRGs could clearly divide the LUAD samples into two distinct clusters (Figure 2D). In addition, the clinical correlation analysis also demonstrated that cluster B patients were positively associated with mild clinical stages including integrated pathological and TMN stages (Figure 2E, Supplementary Table S6) and most of these patients accepted locoregional surgical treatment rather than metastatic surgery or radiation therapy (Figure 2F). All these results suggested that cluster B might be considered as beneficial subtypes with a better prognosis for LUAD patients.

GSVA demonstrated that the immune-related pathways were significantly activated in cluster B patients compared with cluster A groups, including the chemokine signaling pathway, natural killer cell-mediated cytotoxicity, JAK-STAT signaling pathway, and T/B cell receptor signaling pathway (Figure 2G, Supplementary Tables S7, 8). To further explore the immunological characteristics of different subtypes, we performed a series of immune-related analyses including TME, ICI, and immune check-point analyses. For the immune infiltration scores, adaptive immune response-associated lymphocytes (including activated memory CD4^+^ T cells, CD8^+^ T cells, plasma cells, and M1 macrophages) were significantly increased in tissues from cluster B patients than those of cluster A cohorts while regulatory T cells (Tregs) were increased in cluster A patients (Figure 2H, Supplementary Table S10). In terms of TME scores, higher immune scores and stromal scores with lower tumor purity were also observed in patients of cluster B than in the cluster A subtype (Figure 2I, Supplementary Table S9). Interestingly, higher expression levels of immune check-points were detected in cluster A patients than in the other cluster, suggesting its potential therapeutic response to immunotherapies although with severe clinical phenotypes and poor prognosis (Figure 2J).

### Construction and Development of NRG Scores for the Prognosis of Lung Adenocarcinoma

**FIGURE 3:**
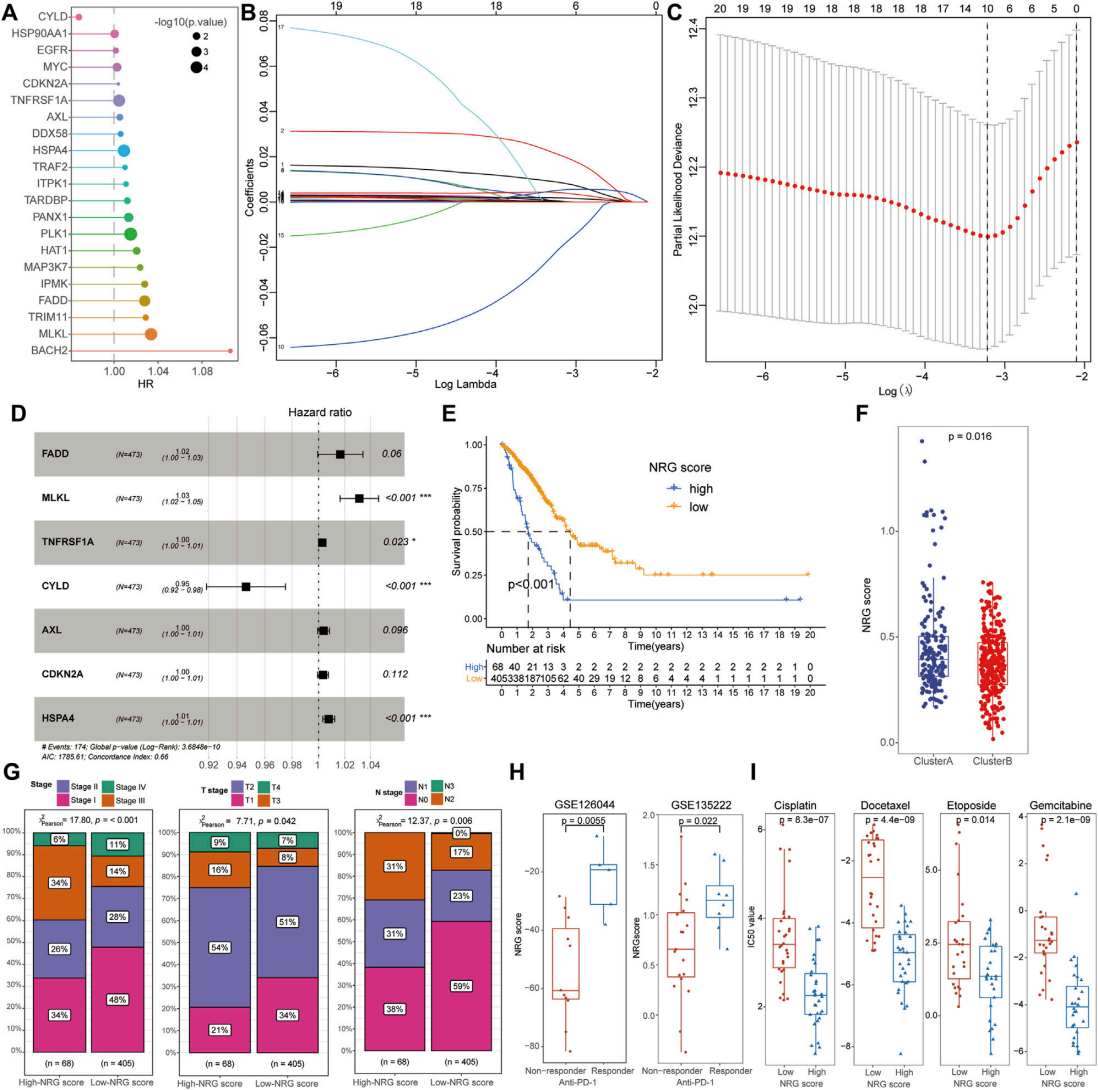
Identification of NRG scores and clinical characteristics in LUAD. **(A)** After univariate Cox regression, 21 signatures were screened as candidate prognosis-associated genes. **(B,C)** Ten NRGs were chosen with the LASSO regression analysis. **(D)** NRG score was defined by seven PRGs (FADD, MLKL, TNFRSF1A, CYLD, AXL, CDKN2A, and HSPA4) with multivariate Cox regression analysis. **(E)** Low-NRG-score patients exhibited a longer survival time than patients with high NRG scores. **(F)** Cluster B patients possessed a lower NRG score than that of cluster A cohorts. **(G)** Low-NRG-score patients exhibited more mild clinical stages than high-score groups. **(H)** NRG scores were significantly increased in patients with an effective response rate to immunotherapy. **(I)** Comparison of IC_50_ value between high- and low-NRG-score patients for common chemotherapeutic drugs including Etoposide, Cisplatin, Gemcitabine, and Docetaxel.

After including the 67 NRGs into the univariate Cox regression, 21 signatures were screened as candidate prognosis-associated genes for the subsequent LASSO and multivariate Cox regression analysis (Figures 3A–C). A total of seven NRGs (FADD, MLKL, TNFRSF1A, CYLD, AXL, CDKN2A, and HSPA4) were successfully identified to construct a novel index representing the characteristics of necroptosis, based on their expression and corresponding *β* coefficients. The NRG score was defined by the following formula: ${\text{NRG}}_{\text{score}}=0.016\times FADD+0.031\times MLKL+0.003\times TNFRSF1A-0.055\times CYLD+0.004\times AXL+0.003\times CDKN2A+0.008\times \text{HSPA}4$ . Subsequently, those LUAD patients were divided into a low- and high-NRG-score subgroup with the optimal cut-off value (0.557) using the “surv_cutpoint” function. Notably, of these hub genes, only CYLD was the protective signature and the high-NRG-score patients exhibited a worse survival state than that of low-score cohorts in TCGA datasets (Figures 3D, E). In addition, we also detected that cluster B groups had lower NRG scores and these low-score patients exhibited a better clinical stage than in the high-NRGscore patients (Figures 3F, G). From both GSE126044 and GSE135222 datasets, those LUAD patients with effective therapeutic responses to anti-PD-L1 therapy exhibited higher NRG scores than those who lacked responses (Figure 3H, Supplementary Table S11). Notably, all these anti-tumor drugs exhibited lower IC_50_ values in the high-NRG-score subgroups, implying that patients with higher NRG scores might gain a better curative effect from classical chemotherapy treatments (Figure 3I, Supplementary Table S12).

### Correlation of Immunological Characteristics and NRG Scores

**FIGURE 4:**
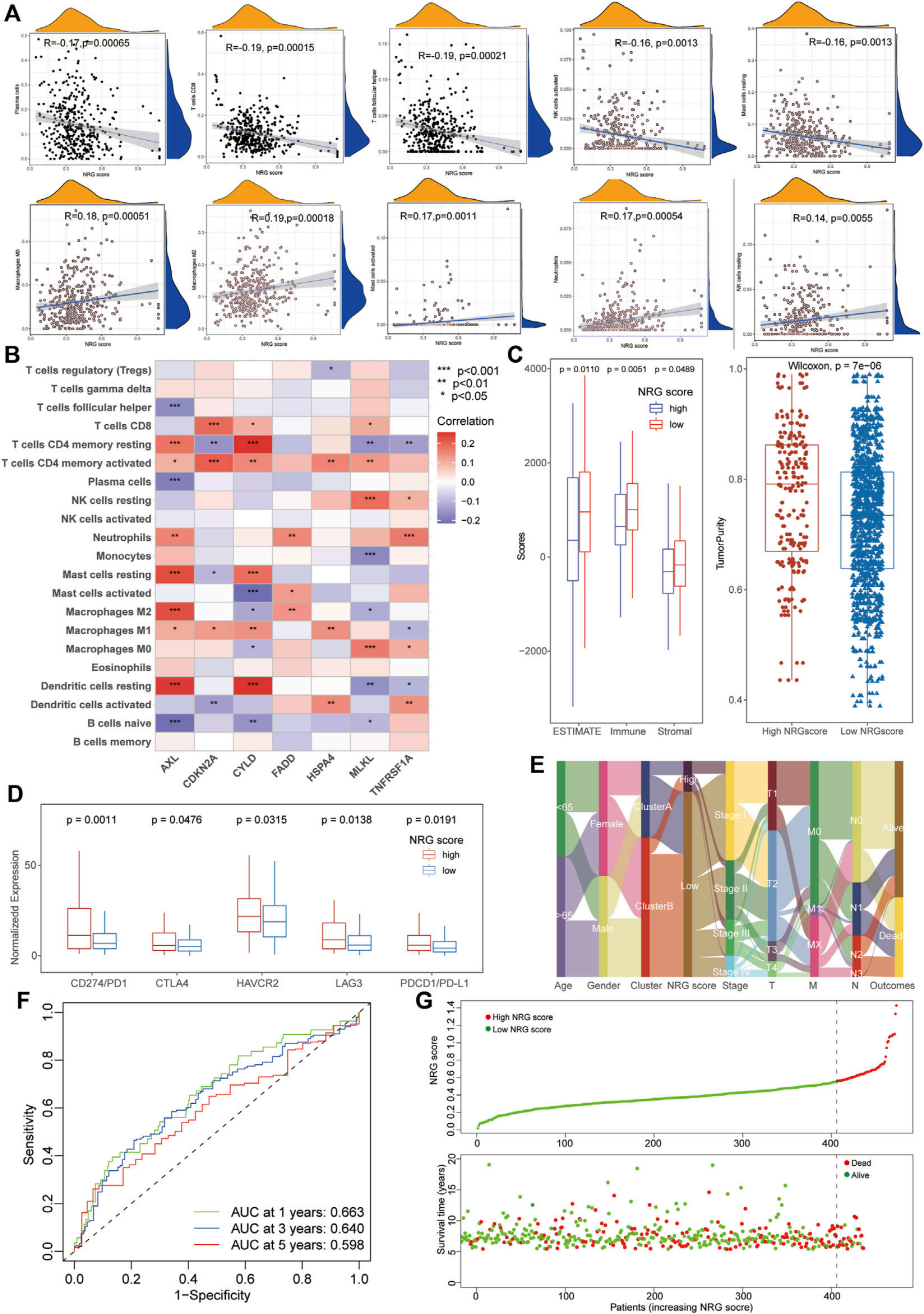
Relationship between immunological characteristics and NRG scores. **(A)** Correlation analysis showed a significant negative correlation between immune cell infiltration and NRG scores. **(B)** Correlation analysis of immune cells infiltration and necroptosis-related signatures of NRG scores. **(C)** TME analysis showed higher stromal and immune scores with lower tumor purity in patients with low NRG scores. **(D)** High-NRG-score patients exhibited higher expression of immune checkpoints. **(E)** The alluvial diagram visualized the status variability of LUAD patients with different subtypes. **(F)** ROC analysis showing the NRG scores could well predict the survival prognosis with high AUC values (1/3/5-year 0.663/0.640/0.598). **(G)** The risk of death was increased with the increase of NRG scores in LUAD.

To investigate the biological characteristics of the NRG scores, we also performed the aforementioned immunological analysis. It revealed that NRG scores were significantly negative-correlated with the abundance of multiple immune cells, including plasma cells, CD8^+^ T cells, Tfh cells, and activated NK cells (Figure 4A). Moreover, the expression of hub necroptosis-related signatures was also associated with the infiltration of immune cells, especially AXL, CDKN2A, and CYLD (Figure 4B, Supplementary Table S13). As expected, the patients with low NRG scores exhibited higher immune scores and stromal scores with lower tumor purity than the high-NRG-score cohorts in the TME analysis (Figure 4C). The expressions of immune check-points were also congruously decreased in the low-NRG score patients, suggesting the consistency between NRG scores and cluster subtypes (Figure 4D). The alluvial diagram clearly visualized that patients’ status varied with different characteristics and we found that most Cluster B patients were divided into the low-NRG-score cohorts with better clinical stages and prognosis (Figure 4E). All these pieces of evidence conformably indicated that the low-NRG-score patients, consistent with cluster B subtypes, possessed an immune-activated status and better prognosis for LUAD. The ROC analysis further indicated that the NRG scores could well predict the survival prognosis with high AUC values (1/3/5-year: 0.663/0.640/0.598, respectively) and the risk of death was also increased with the increasing of NRG scores in TCGA datasets (Figures 4F, G).

### Relationship Among mRNA Stem Index, Tumor Mutation Burden, and NRG Scores

**FIGURE 5:**
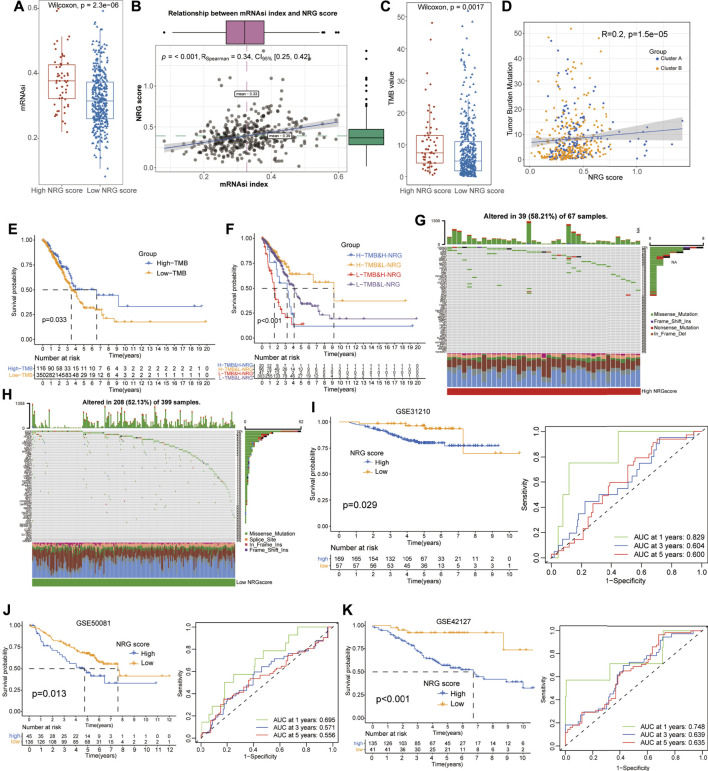
Evaluation of potential therapeutical susceptibility and prognostic value of NRG scores in LUAD. **(A,B)** Low-NRG-score patients exhibited lower levels and a significant positive correlation with the mRNAsi index (R = 0.34). **(C,D)** Low-NRG-score patients exhibited lower levels and a significant positive correlation with TMB values (R = 0.20). **(E)** Survival analysis shows that the high-TMB patients exhibited a better prognosis for LUAD patients. **(F)** Stratified survival analysis revealed that patients with low NRG scores and high TMB values had the best prognosis status for LUAD. **(G,H)** Condition of somatic mutation of NRGs in high- and low-TMB patients. **(I–K)** Low-NRG-scores patients displayed a better prognosis and NRG scores could accurately estimate the OS for LUAD in external datasets (1- /3- /5-year AUC values: 0.829/0.604/0.600 in GSE31210; 0.695/0.571/0.556 in GSE58001; and 0.748/0.639/0.635 in GSE42127).

As a novel prognosis indicator in oncological studies, the mRNAsi score was obtained from Tathiane’s article (Malta et al., 2018), and the TMB score was calculated based on the mutation data of LUAD patients from TCGA datasets, which represent their correlation with curative effects and prognosis multiple tumors. To further investigate their potential relationships with NRG scores, we compared the TMB and mRNAsi levels between different NRG score subgroups and conducted the correlation analysis with Spearman’s methods (Supplementary Tables S14, 15). The results revealed that the low-NRG score patients exhibited lower mRNAsi and TMB scores than that of high-NRG-score cohorts (Wilcox test, *p* = 2.3e−06, *p* = 0.0017, Figures 5A, C) and the NRG scores were positively associated with mRNAsi and the TMB index (Spearman coefficient: *R* = 0.34, *p* < 0.001; *R* = 0.20, *p* = 1.5e−05, Figures 5B, D). Notably, the survival analysis detected a longer median survival time in high-TMB-score patients than those in low-score patients, contradictory with the better prognosis in low-NRG-score patients (Figure 5E). To interpret this conflict, we performed stratified survival analysis and observed that patients with high-TMB and low-NRG scores exhibited the best prognosis status and NRG scores played a more effective role in predicting the prognosis than TMB scores, suggesting the independent effects of NRG and TMB scores for the prognostic stratification of LUAD (Figure 5F). Moreover, we also evaluated the distribution of somatic variants between the high- and low-NRG-score cohorts using TCGA datasets and multiple mutation patterns were detected in both subgroups including missense mutation and frame shift ins. Interestingly, there was no significant difference in the mutation frequency between the two subgroups (58.21% in high-NRG vs. 52.13% in low-NRG patients) but CDKN2A, HDAC9, and ALK were the top three NRGs with the most mutation frequency in high-NRG patients while EGFR, BRAF, and ATRX were the top three NRGs in low NRG cohorts (Figures 5G, H).

### Evaluation and Validation of the Prognostic Model for Lung Adenocarcinoma

**FIGURE 6:**
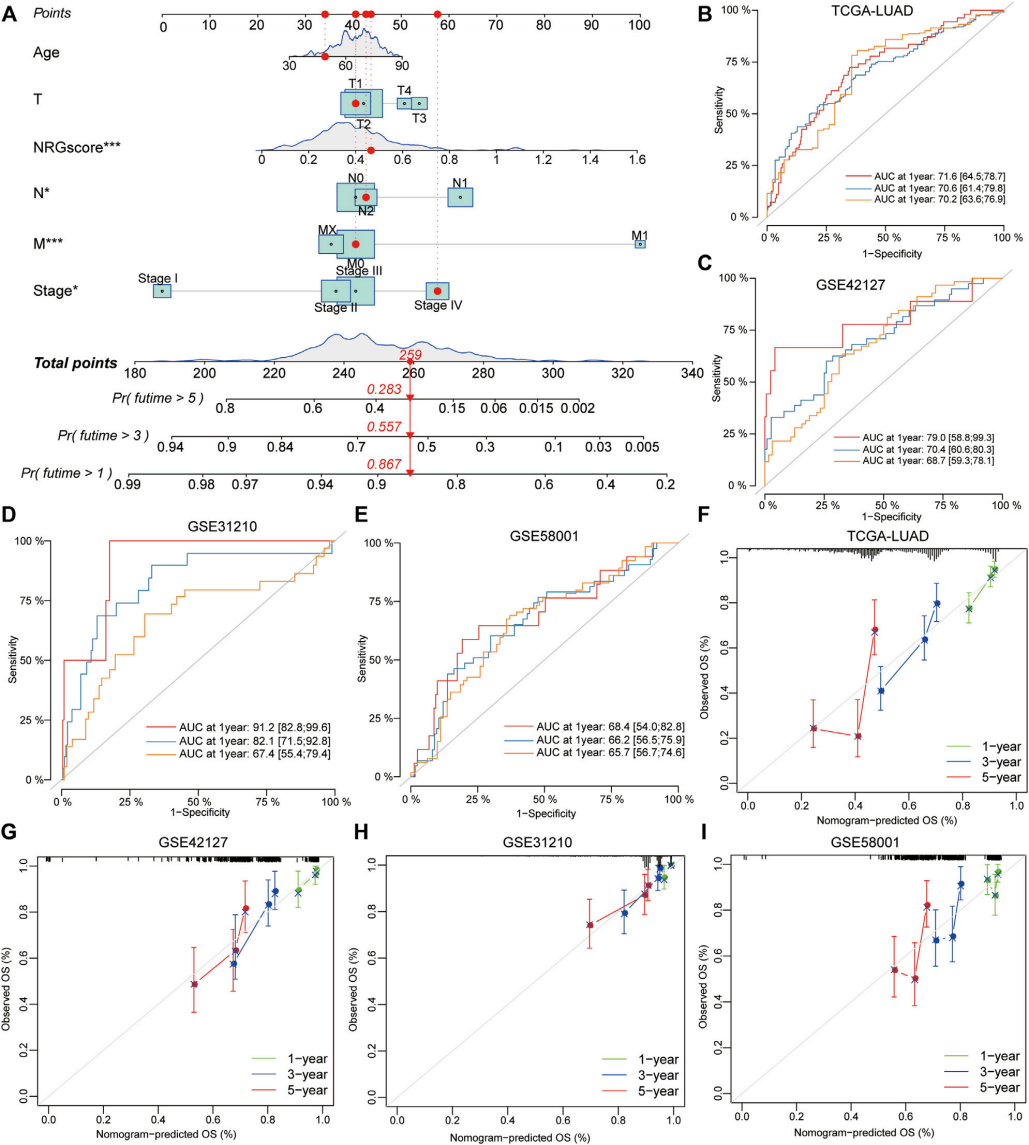
Development and validation of a prognostic model for LUAD patients. **(A)** Combined nomogram for predicting the probability of 1/3/5-year survival for LUAD patients, based on age, clinical stages, TNM stages, and NRG scores. **(B–E)** ROC analysis showed a good prediction capacity for LUAD patients with high mean AUC values: (0.716/0.706/0.702) in TCGA datasets, 0.790/0.704/0.687 in GSE42127, 0.912/0.821/0.674 in GSE31210, and 0.684/0.662/0.657 in GSE58001 datasets. **(F–I)** Calibration curve of the established nomogram with 1/3/5-year survival, respectively.

To validate the prognostic value of NRG scores in LUAD, other three external GEO datasets were applied to perform the survival and ROC analyses. Notably, it revealed that low-NRG-score patients displayed a better prognosis, and NRG scores could accurately estimate the overall survival for LUAD in all datasets (1- /3- /5-year AUC values: 0.829/0.604/0.600 in GSE31210; 0.695/0.571/0.556 in GSE58001; and 0.748/0.639/0.635 in GSE42127, respectively) (Figures 5I–K, Supplementary Tables S16). Subsequently, based on the NRG scores and other important clinical features, the nomogram was successfully constructed using the multivariate Cox model to predict 1/3/5-year survival rates for LUAD patients. Age, clinical stages, TNM stages, and NRG scores were included in the nomogram (Figure 6A), and the calibration curve showed a good prediction capacity for LUAD patients with high mean AUC values (0.716/0.706/0.702) in TCGA datasets (Figures 6B, F). In addition, the external datasets further demonstrated the predictive capability of the nomogram for the prognosis in LUAD patients, including 0.790/0.704/0.687 in GSE42127, 0.912/0.821/0.674 in GSE31210, and 0.684/0.662/0.657 in GSE58001 datasets (Figures 6C–E, G–I).

## Discussion

As one of the malignant tumors with high mortality, the outcome of LUAD patients remains poor because of the lack of effective therapeutical responses to chemotherapy and immunotherapy due to inner biological heterogeneity (Yuan et al., 2021). Over the past decades, the identification of histological subtypes with an especial genic mutation has brought dramatic amelioration in disease outcomes of LUAD patients. In particular, massive molecularly targeted anticancer agents, including EGFR and ALK inhibitors, have been approved as the preferred treatments for LUAD patients with corresponding genetic alterations (Gridelli et al., 2014). Moreover, immune checkpoint genes (such as PD1/PD-L1, LAG-3, CTLA-4, and HAVCR2) have been certified to participate in the immune suppression process of multiple tumors and targeted inhibitors have also been applied to specific immunotherapy for cancers (Zhang H. et al., 2021). However, in our clinical practice, even if the LUAD patients were at the same pathological stages, their therapeutical response to the targeted immunotherapy might still be completely different (Skoulidis and Heymach, 2019). Therefore, it is urgently required to identify a novel molecular subtype and reliable prognostic model for predicting the outcomes of LUAD patients.

Different from cellular apoptosis, necroptosis has been recognized as a specially programmed cell death with an essential role in maintaining the stabilization of the internal environment and participating in the pathogenesis of multiple diseases including various infections, tumor formation, and autoimmune diseases (Schreiber et al., 2017; Wang et al., 2019; Xia et al., 2020). Increasing studies have identified necroptosis-related gene signatures and subtypes to predict the prognosis and therapeutic response of multiple tumors including breast cancer (Chen W. et al., 2022), kidney renal clear cell carcinoma (Chen W. et al., 2022), and pancreatic adenocarcinoma (Wu et al., 2022), but there is no research focusing on the relationships between NRGs and LUAD.

Various molecular genetic alterations have provided valuable information for predicting the risk and prognosis of LUAD patients, especially based on copy number variations (CNVs) and somatic mutation analysis (Zhao Y. et al., 2021). In this study, we also explored the genetic characteristics of NRGs in LUAD patients and it revealed that a high somatic mutation frequency (52.76% samples) was detected and most NRGs possessed copy number amplification, suggesting that necroptosis might be closely associated with a genetic mutation in LUAD patients. The classification of LUAD patients based on various biological signatures has been considered a promising method and applied to various studies including immune cell infiltration (ICI) (Li et al., 2021), autophagy (Zhang M.-Y. et al., 2021), pyroptosis (Dong et al., 2021), and m6A RNA methylation (Zhou et al., 2021). Therefore, this study first proposed a necroptosis-related subtype for LUAD based on the clustering expression of NRGs with distinct prognostic and immunological features including TME, ICI, GSVA, and immune checkpoints. Notably, cluster B patients exhibited a longer median survival time than cluster A cohorts, and the prognostic clusters were consistent with clinical TNM stages, indicating that these NRGs were significantly related to survival status in LUAD patients. In addition, the TME analysis detected higher immune and stromal scores in cluster B than in cluster A and GSVA also detected the activation of immune-related pathways in cluster B cohorts, suggesting that the anticancer immune response was significantly activated in cluster B patients. Immune checkpoint genes (including PD1, PD-L1, CTLA4, HAVCR2, and LAG3) have been demonstrated to play an essential role in the immune suppression of multiple cancers and several targeted inhibitors, especially PD1/PD-L1, and have also been widely applied to clinical immunotherapy for tumors (Kim and Choi, 2020). Interestingly, the expression of these immune checkpoints was significantly decreased in cluster A patients suggesting a significant immune exhaustion status and a possible better therapeutical response in LUAD. All these results indicated that cluster B was an immune-activated subtype with a better prognosis and potential curative response for LUAD cohorts.

Furthermore, a novel necroptosis-related tool (NRG score) was successfully identified to estimate the prognostic risk of LUAD patients based on the stepwise model of multivariate Cox regression. Interestingly, a better survival status with lower TNM stages, higher TME scores, and a significant negative correlation with ICI scores were also detected in low-NRG score subgroups, consistent with the characteristics of cluster B patients. Patients with high NRG scores also exhibited a higher expression of immune checkpoints than low-NRG-score patients, indicating their potential therapeutic sensitiveness to immunotherapy for LUAD. Interestingly, the NRG score was calculated based on the expression of hub NRGs, especially MLKL, HSP4A, and CYLD, all associated with the pathogenesis and prognosis of LUAD by previously published works. As the executor of necrotic apoptosis, MLKL had been reported to be activated by RIPK1 or RIPK3 with phosphorylation to mediate necrosis signaling and play an important role in various non-necroptotic processes including receptor internalization, ligand-receptor degradation, axonal repair, and necroptotic inhibition (Brault and Oberst, 2017; Martens et al., 2021). *In vitro*, Tan et al. (2020) further demonstrated that the activation of RIP3/MLKL-dependent necroptosis could increase the therapeutic sensitivity to gefitinib in NSCLC patients. Heat-shock protein family A (Hsp70) member 4 (HSPA4) was involved in the functional stabilization of mutated or aberrantly expressed genes in multiple tumors (Lv et al., 2012) and had been identified to have a significant correlation with immune regulation and prognosis of hepatocellular carcinoma (Shang et al., 2021). Notably, as the sole protective NRG for LUAD, CYLD Lysine 63 Deubiquitinase (CYLD) had been considered as the tumor suppressor and further demonstrated to be regulated by miR-96-5p and LncRNA GMDS-AS1 to inhibit the development of LUAD via a cellular assay and mouse tumor models (Zhao et al., 2020). These results indicate the NRG’s potential relationship with the prognosis of LUAD and the specific mechanism of these vital signatures in LUAD remains to be further explored by functional experiments *in vivo* or *in vitro*.

Cancer stem cells (CSCs) are characterized by unlimited proliferation and self-renewal and have participated in the therapeutic resistance of lung cancers. As the most representative parameter of CSCs, the mRNA stem index (mRNAsi) has been widely applied to evaluate the characteristics of CSCs and prognosis in a variety of tumors including LUAD (Zhang et al., 2020). The TMB value has been identified as a novel biomarker of response to immune checkpoint treatment and reported to predict the survival status of LUAD patients (Nan et al., 2021). Therefore, the mRNAsi index and TMB value could serve as sensitive indexes to the response of immunotherapy. In this study, we also performed the correlation analysis of the mRNAsi index, TMB value, and NRG scores, and our results exhibited that there was a significant positive relationship among the mRNAsi index, TMB value, and NRG scores, consistent with the aforementioned finding of immune checkpoint expression. Moreover, the stratified survival analysis demonstrated that the prognosis capability of NRG scores was independent of the TMB value and those patients with low NRG and high TMB possessed an optimal survival status. It was worth noting that patients with low-NRG scores could still exhibit favorable survival regardless of different TMB conditions, suggesting that NRG scores might be a more effective predictor than TMB values. More importantly, based on the datasets with immunotherapy, we successfully validated the potential relationship between NRG scores and clinical response to immunotherapy in LUAD patients. Common chemotherapeutic drugs also exhibited lower IC_50_ values in high-NRG-score patients, including Etoposide, Cisplatin, Gemcitabine, and Docetaxel, indicating a more effective role in LUAD patients with high NRG scores.

Furthermore, to validate the significance of NRG scores in predicting the prognosis of LUAD, other external GEO datasets including 561 patients were used to perform the ROC analysis and we found that NRG scores actually predicted 1/3/5-year survival outcomes of LUAD with high mean AUC values. Combined with age, clinical stages, TNM stages, and NRG scores, we successfully constructed a novel nomogram tool to accurately predict the 1-, 3-, and 5-year OS probability of individual LUAD patients. More importantly, the predictive capability of the nomogram was successfully validated through the external GEO datasets based on calibration curves and ROC curves, implying the stabilization of the model in LUAD.

However, there are still several ineluctable limitations in our study. On the one hand, the integrated analysis based on the transcriptomic profiles was only obtained from public open-source databases and the size of LUAD cohorts in the databases was relatively small and limited. Therefore, some corresponding results, such as necroptosis-related subtypes, remain to be further validated via more external self-sequencing datasets or experiments *in vivo* and *in vitro*. On the other hand, further application of NRG scores still needs other fundamental studies and even clinical practices to be reduplicatively validated and ameliorated. Finally, the complicated mechanism of NRGs in the development of LUAD was still unclear and needed to be further deeply explored through experiments *in vivo* or *in vitro*.

## Conclusion

In conclusion, this study first identified a necroptosis-related disease subtype based on the unsupervised clustering of NRGs with different clinical and immunological signatures in LUAD patients. Furthermore, we also defined a promising tool called the “NRG score” to predict the OS status and potential therapeutic response to immunotherapy for LUAD. Finally, combined with age, clinical stages, TNM stages, and NRG scores, we successfully constructed a novel nomogram tool to accurately predict the 1-, 3-, and 5-year OS probability of individual LUAD patients, and this model was favorably validated in multiple external GEO datasets with concordant calibration curves and high AUC values. Integrated transcriptomic analysis helps us seek vital necroptosis-related genes and supplements a novel clinical application of NRG scores in predicting the overall survival and therapeutic benefits for LUAD patients.

## Data Availability

The original contributions presented in the study are included in the article/Supplementary Material; further inquiries can be directed to the corresponding author.
