# Supplementary figures and images for: Characterization of Necroptosis-Related Molecular Subtypes and Therapeutic Response in Lung Adenocarcinoma
Source: Front Genet. 2022 Jun 8;13:920350. doi: 10.3389/fgene.2022.920350 (PMC9214237; doi:10.3389/fgene.2022.920350)

Supplementary Figure 1

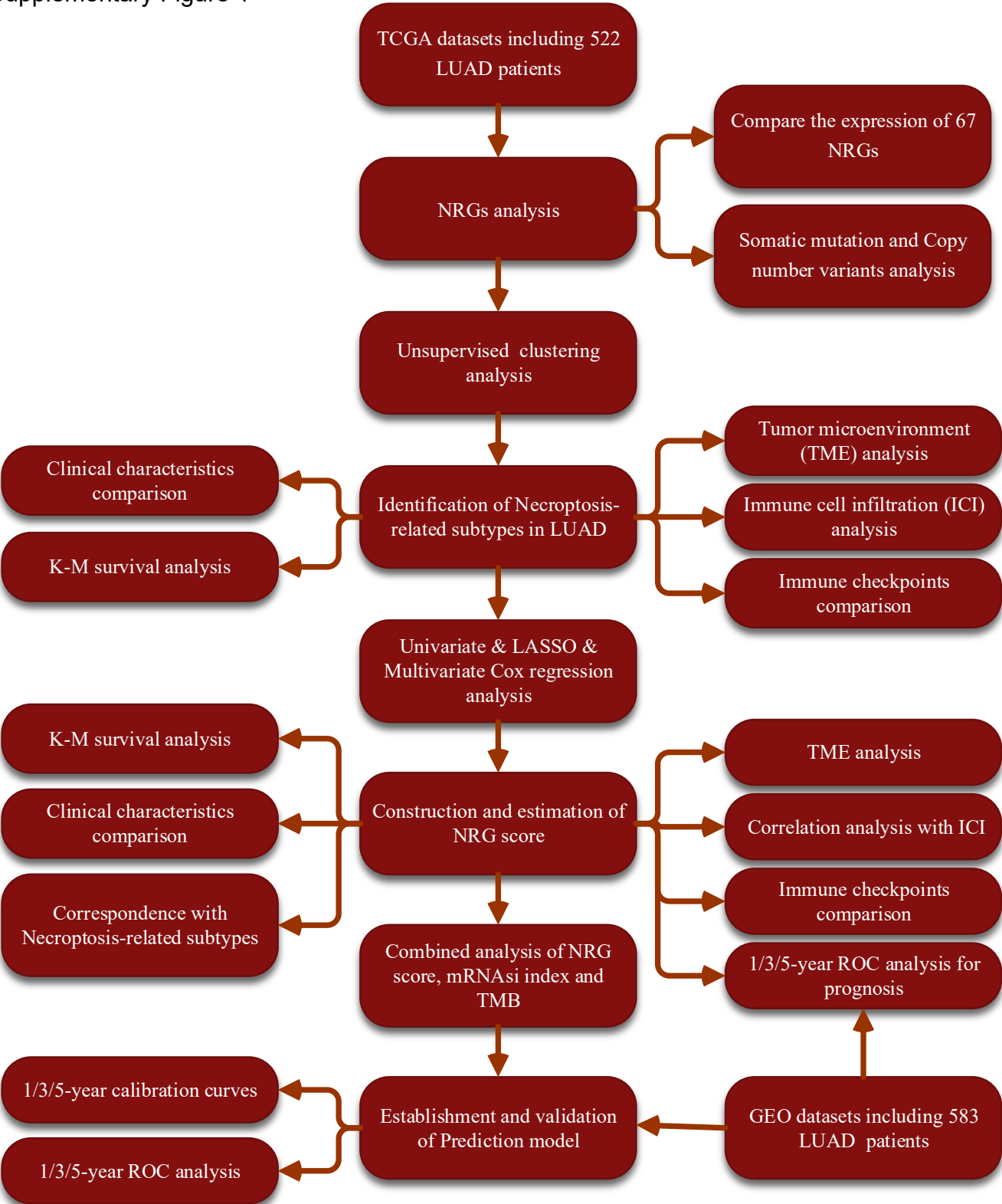

Supplement: Supplementary file 1 [file Image1.pdf]
